# Linear Population Allocation by Bistable Switches in Response to Transient Stimulation

**DOI:** 10.1371/journal.pone.0105408

**Published:** 2014-08-20

**Authors:** Jaydeep K. Srimani, Guang Yao, John Neu, Yu Tanouchi, Tae Jun Lee, Lingchong You

**Affiliations:** 1 Department of Biomedical Engineering, Duke University, Durham, North Carolina, United States of America; 2 Department of Molecular & Cellular Biology, University of Arizona, Tucson, Arizona, United States of America; Semmelweis University, Hungary

## Abstract

Many cellular decision processes, including proliferation, differentiation, and phenotypic switching, are controlled by bistable signaling networks. In response to transient or intermediate input signals, these networks allocate a population fraction to each of two distinct states (e.g. OFF and ON). While extensive studies have been carried out to analyze various bistable networks, they are primarily focused on responses of bistable networks to sustained input signals. In this work, we investigate the response characteristics of bistable networks to transient signals, using both theoretical analysis and numerical simulation. We find that bistable systems exhibit a common property: for input signals with short durations, the fraction of switching cells increases linearly with the signal duration, allowing the population to integrate transient signals to tune its response. We propose that this allocation algorithm can be an optimal response strategy for certain cellular decisions in which excessive switching results in lower population fitness.

## Introduction

To optimally survive, cell populations must appropriately respond to changes in environmental conditions, such as availability of nutrients, presence of competition, and perturbations by drugs. These environmental cues often trigger distinct cell fate responses (e.g., rest vs. grow; live vs. die) in an otherwise homogeneous cell population. These distinct responses are important to both the fitness of unicellular microbes and the proper cellular functions in multicellular organisms, e.g., tissue homeostasis [1], differentiation [2], and wound healing [3]–[5].

Despite the diversity of cell fate response systems, many share a common property: the phenotypic response is controlled by a bistable switch that assumes one of two stable states (e.g. OFF and ON). Extensive modeling and experimental studies have been carried out to understand the generation and modulation of the bistable systems, primarily on their responses to sustained signals [6], [7]. However, bistable switches can operate under conditions where the input signals are transient. For example, *Bacillus subtilis* shifts from vegetative growth to competence and sporulation in a nutrient-limited environment [8]. It reverts to the vegetative state when nutrient is available. If too many individuals revert, the nutrient level may be insufficient to support them; if too few individuals revert, the population would fail to fully utilize the available nutrients. It turns out that the fraction of reverted individuals is dependent on the nutrient duration, in a process controlled by a bistable switch involving the regulatory gene *SpoA* [9].

Bistable decisions also underlie the development and maintenance of virulence factors in a variety of bacterial pathogens, including the human pathogens *Salmonella typhimurium* [10], [11] and *Pseudomonas auruginosa* [12], as well as the plant pathogen *Dickeya dadantii* [13]. A major driver of virulence in all these species is the expression of a Type III secretion system (T3SS) that allows the pathogen to inject host cells with protein factors that enable invasion [14]. Single-cell studies have shown that the T3SS is often expressed in a bistable manner in response to environmental factors such as salt concentrations [10]. That is, a clonal population contains a mix of two phenotypic subsets, T3SS+ and T3SS−. While the T3SS+ subset enables the *Salmonella* population to invade the host [15], it has a slower growth rate (probably due to an increased metabolic burden and the presence of gene regulons that couple metabolism and virulence) [16], [17]. Therefore, a tradeoff is necessary between growth and virulence – if the T3SS+ fraction is too high, the population cannot multiply fast enough to withstand host defense mechanisms; if the T3SS+ fraction is too low, the population cannot invade the host cells effectively. To this end, the bistable system that controls the T3SS expression may coordinate the population distribution (T3SS+ vs. T3SS−) based on the favorability of environmental conditions.

Another example is bacterial programmed altruistic death (PAD). In response to stress, a fraction of a population undergoes programmed death, which leads to the release of certain “public good” molecules and ultimately the stress alleviation, thus benefiting the survivors [18], [19]. For each cell, death is a bistable decision (a cell either dies or lives). We have previously shown that an optimal death rate exists for a given stress level. If the death rate is too low, the population will be unable to alleviate the stress; if the death rate is too high, the population will be unable to recover, even after the removal of stress [20]. When a PAD-capable population is subjected to transient stress, such as periodic antibiotic dosing, the bistable network might inherently set the optimal death rate, based on the dosing profile.

**Figure 1:**
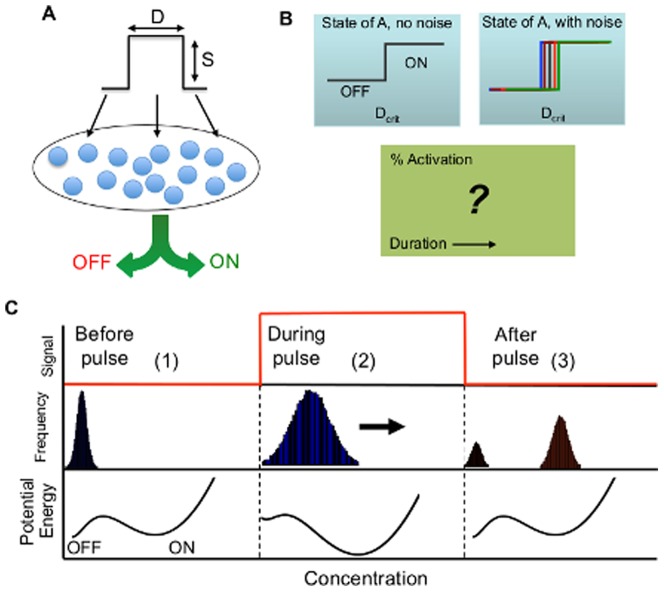
Stimulation of a bistable network by a pulse input. (A) A pulse input can be specified by its *intensity (S)* and *duration (D)*. Stimulation of a stochastic bistable network in an isogenic population using this pulse results in the emergence of two distinct subpopulations (e.g. OFF and ON). (B) If the activation is deterministic, there exists a critical pulse duration beyond which all individuals in a population will transition between the two steady states (e.g. from OFF to ON) (top panel). Presence of noise will result in variability in the critical duration: different individuals will be activated at different thresholds (middle panel). For a short duration, a fraction of a population will be allocated to either steady state. Here, we aim to investigate this population activation, which we term “activation probability”, as a function of both pulse intensity and duration (bottom panel). (C) Schematic shows the evolution of a population of cells carrying a bistable switch (blue) in response to a pulse input (red). Potential energy landscapes are shown in black. In the absence of stimulus (1), the population is in the OFF state. When stimulus is applied (2), the potential energy landscape shifts in favor of the ON state, driving the population toward the ON state. When the stimulus is removed (3), the potential energy landscape reverts to its initial state, splitting the population into two subpopulations (OFF and ON).

Given these phenomena, we wondered how a bistable switch responds to transient signals. Without loss of generality, each transient signal can be approximated by a rectangular pulse (Figure 1A) with intensity *S* and duration *D*. If the switch operates in the absence of noise (in a hypothetical cell population), we expect a uniform, binary transition (e.g. from OFF to ON) at a critical stimulus duration, *D_crit_*, which is a function of the switch parameters and the signal intensity *S*. However, in the presence of cellular noise, *D_crit_* may vary from cell to cell (Figure 1B). The superimposing of stochasticity onto bistability leads to the division of a cell population into two subpopulations, e.g. OFF and ON. Intuitively, the magnitude of stochastic noise must be restricted within some intermediate range to ensure the emergence of a bimodal population: at extremely low noise levels, we would expect to see a deterministic transition with increasing stimulus. In contrast, excessively high noise would overwhelm the network dynamics.

Here we examine *how the fraction of activated individuals (the ON subpopulation) depends on the strength and duration of the transient input signals*. We use theoretical analysis and computational simulations to investigate the input-output function of three representative bistable systems with increasing complexity. In particular, we focused on the effects of varying input signal parameters and noise levels. We show that increasing the stimulus duration results in an exponential asymptotic increase in population activation. When the input duration is short, this dependence is *linear*, and this property is maintained for all three bistable systems. We propose that this linear dependence (activated fraction vs. signal duration) can function as a survival strategy, representing an optimal response to transient input signals. We use a minimal model to demonstrate that when pulse (e.g. nutrient) duration governs growth rate, over-allocation results in slower growth due to cells having fewer resources; conversely, under-allocation results in wasted excess resources. A linear strategy balances these two detrimental effects, enabling populations to proliferate faster than either extreme. In this regard, a bistable switch acts as an integrator, enabling a cell population to assess transient signals based on intensity and duration and respond accordingly.

## Results

### A positive-feedback circuit

**Figure 2:**
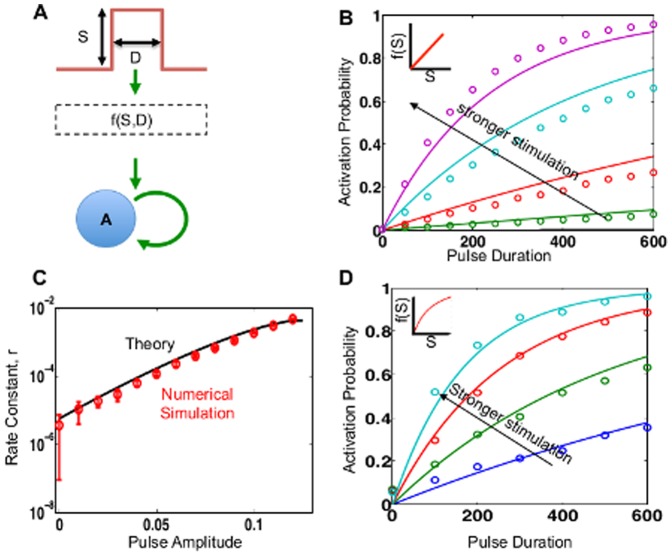
Response of a positive-feedback model to pulse inputs. (A) Triggering a bistable positive-feedback model with a pulse. (B) Simulation (open circles) vs. theoretical prediction (solid lines) of activation probability as a function of pulse duration. The black arrow indicates increasing stimulus intensity. The activation probability increases linearly with the duration, when the latter is small. Inset shows intermediate signal transformation function. (C) The transition rate dictates the linear dependence, and increases with increasing stimulus intensity. Open circles and lines indicate theoretical and numerical predictions, respectively. (D) The pulse strength can be monotonically scaled upstream of the bistable decision module without affecting the characteristic activation property. Here, we use a Hill function with coefficient *n* = 2 as the transformation function (shown in inset).

We start with a minimal bistable system consisting of a protein driving its own synthesis, forming a positive feedback loop (Figure 2A). This system can be modeled with a single ordinary differential equation (ODE): 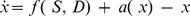. The three terms on the right hand side of the equation, from left to right, correspond to external stimulation, nonlinear positive feedback, and decay, respectively. Without loss of generality, we choose 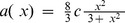 and the parameter *c* such that the system is bistable in the absence of stimulus (*f* = 0), with an unstable steady state at 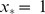, and 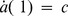. A larger value of *c* corresponds to stronger positive feedback, which increases the distance between the two steady states (Figure S1A). We use the Fokker-Planck formulation of this non-dimensional ODE to incorporate the effects of both mechanism-dependent (intrinsic) and independent (extrinsic) noise sources, which we assume to be Gaussian distributed. This formulation enables us to simulate the noise inherent in biochemical reactions, due to the effects of both discrete small numbers of molecules as well as small reaction volumes (i.e. cells). Unless 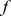 is saturating, the potential landscape resulting from this stochastic ODE has two wells corresponding to two stable steady states (Figure S1B), separated by an energy barrier at the unstable steady state. For saturating 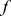, the landscape is monostable.

For 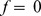, consider a population of cells residing at the OFF state, assuming a Gaussian protein distribution (Figure 1C, panel 1). When the stimulus is applied (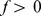), the landscape shifts in favor of the ON state: the energy barrier is lowered and the ON state becomes more stable. This shifted landscape promotes stochastic transition from the OFF to the ON state (panel 2), as if the population of cells are gradually sliding toward the ON state. When the stimulus is turned off, the potential landscape reverts to the initial configuration (panel 3). At this moment, the population will be split into two subpopulations, depending on the relative position of each cell in comparison to the unstable steady state. Cells to the right of the unstable steady state will continue to move toward the ON state; those to the left will move back to the OFF state. The fraction of activated cells (or the activation probability) depends on the transition rate, which in turn depends on stimulus intensity and noise magnitude (Figure S2).

Using the Fokker-Planck equation, we find that, for a given input intensity, the activation probability increases with the input duration, approximately following an exponential asymptotic function: 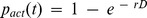. The transition rate constant, 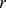, is a function of the input strength (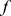) and noise magnitude (see Supplemental Information for full derivation). This theoretical solution agrees well with results from numerical simulations, using the corresponding stochastic differential equations (SDE) (Figure 2B). As expected, stronger stimuli result in a deeper ON well, and a faster increase in activation probability, characterized by larger 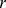 values (Figure 2C). Importantly, for sufficiently small 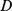, 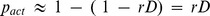. That is, for a population of cells exposed to a short stimulus, the number of cells that switch increases linearly with the signal duration.

In general, a pulse input can be processed by an intermediate signaling module or cascade in the cell, prior to the bistable module. Mathematically, this processing can be considered as a transform by a relevant function (e.g. a Hill function). The resulting scaled stimulus will assume a modified shape dictated by the signaling module throughout the stimulus duration, while remaining zero for 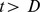. As long as the transformation is monotonic in terms of signal strength, it does not qualitatively change the allocation property (Figure 2D, inset shows intermediate transformation): the activation probability increases approximately linearly with increasing duration of the input pulse, when the duration is small.

Our analysis confirms previous studies of switching properties, which have described exponentially distributed residence times in potential wells using Fokker-Planck formulations of population drift and diffusion [21]–[24]. However, these studies utilize arbitrary two-well potential functions to realize transitions from one state to another. It is unclear to what extent these conclusions are applicable to more complex bistable systems, based on molecular mechanisms, which exist in nature. Furthermore, these studies do not address the potential biological implications of these switching properties. To that end, we use numerical simulations to interrogate the response of two increasingly complex bistable frameworks, and describe a general biological scenario in which conserved switching properties may be advantageous to bistable populations.

### A toggle switch model

**Figure 3:**
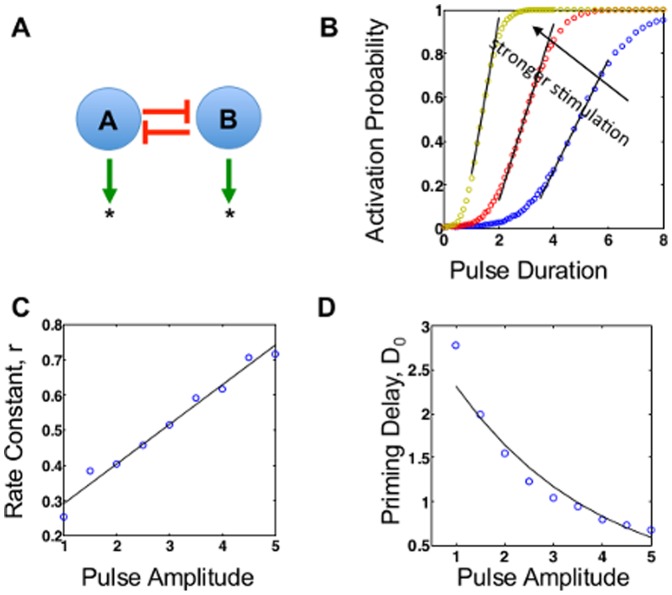
Response of a toggle-switch to pulse inputs. (A) A toggle switch consisting of two molecules (A and B) inhibiting each other. With sufficient nonlinearity, the system is bistable, with either A or B dominating. Our analysis assumes that all cells are initiated at state A. A pulse input is applied to drive them into state B. (B) Simulated dependence of the activation probability on pulse duration. For sub-saturating durations, the dependence exhibits an approximately threshold-linear property: there is no activation until the pulse duration is above a threshold; then the activation probability increases linearly (as shown by solid lines, R^2^>0.9) until saturation. Black arrow indicates direction of increasing stimulus intensity. (C) The linear activation regime slope *r* increases with stimulus intensity (linear fit, R^2^ = 0.987). (D)The priming delay *D*_0_ decreases with stimulus intensity (exponential decay R^2^ = 0.972).

To test the general applicability of the linear response, we next examine the response of a toggle switch [25] to transient stimulation. This motif has been found in numerous natural biological networks [26], [27], and is one of the first regulatory motifs to be implemented and analyzed with synthetic gene circuits using different genetic elements, in bacteria, yeast, and mammalian cells [28]–[31]. The toggle switch consists of two molecular species mutually inhibiting each other (Figure 3A). Due to its greater complexity, we employ dimensionless numerical simulations using an SDE model (all units are arbitrary, see SI for details) to examine its response to a pulse input.

Our simulation results show that the toggle switch exhibits a “priming” period (*D*_0_): for signal durations less than this value, the switch is minimally activated. This feature is likely due to the inhibitory mechanism. For example, assuming a population is initially in state A (Figure 3A), weak upregulation of B (short duration) will be unable to overcome the inhibition of B imposed by high levels of A. For stimuli longer than *D*_0_, the system follows the same short duration linear approximation as the cooperative model. We can fit the activation probability curve using 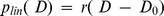, taking 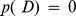 for 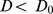. A representative example is shown in Figure 3B (solid line, see Materials and Methods for details). In addition, our results show that the rate of linear increase, *r,* is positively correlated with the signal intensity (Figure 3C, solid line shows linear fit, R^2^ = 0.987) and that 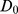 should decrease with increasing stimulus intensity, as shown in Figure 3D (solid line shows exponential decay, R^2^ = 0.972). Here, it is important to note that a priming period may occur for any bistable motif, given sufficiently short pulse duration. That is, if the pulse duration is short enough that activation is outweighed by degradation, the system would experience a priming delay. In the preceding positive feedback model, the lack of inhibition means that the priming delay is negligibly short compared to the subsequent linear activation region.

### A bistable Myc-Rb-E2F model

**Figure 4:**
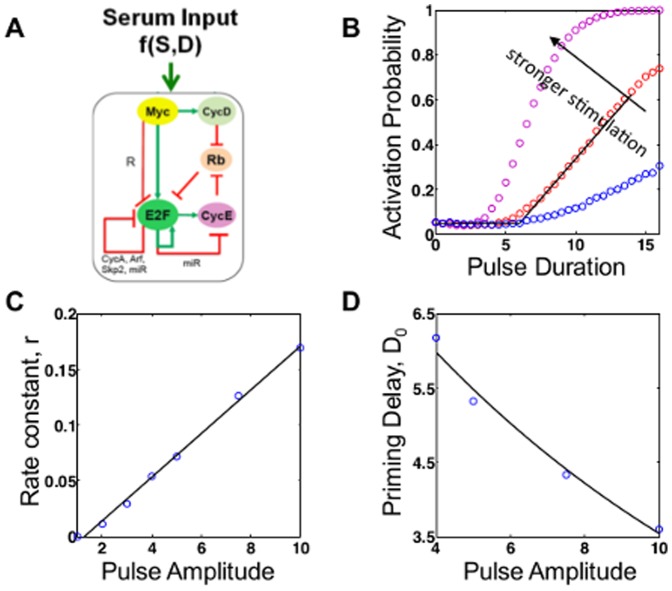
Response of the Myc-Rb-E2F network to pulse inputs. (A) In response to serum stimulation, the Myc-Rb-E2F can exhibit bistability in E2F levels. (B) Simulated dependence of activation probability on the duration of the pulse input. Solid black arrow indicates increasing stimulus intensity. The dependence is approximately threshold linear for sub-saturating durations. Black arrow indicates direction of increasing stimulus intensity. (C) The linear activation regime slope *r* increases with stimulus intensity (linear fit, R^2^ = 0.997). (D) The priming delay *D*_0_ decreases with stimulus intensity (exponential decay R^2^ = 0.985).

We next examine a much more complex network, Myc-Rb-E2F network (Figure 4A), which plays a critical role in controlling cell cycle entry and cell fate decisions [32], [33]. Dysregulation of this network has been shown to contribute to uncontrolled cell proliferation and cancer development. Our previous work [34]–[36] has studied the dynamic network properties underlying E2F-mediated cell cycle entry. In normal cells, serum stimulation can lead to bistable activation of E2F, which in turn serves as a master-regulator of genes involved in cell cycle entry.

Again, we adopt an SDE model [37] of the Myc-Rb-E2F network to examine its response to transient growth stimulation (Figure 4A and Figure S3). Despite its much greater complexity, this model exhibits qualitatively the same trend in activation probability (Figure 4B) as the toggle switch model. In particular, a population exhibits a threshold-linear response: when the stimulation duration is shorter than a threshold value (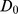 the priming duration), the network does not respond significantly. In this case, the priming delay is due to a number of inhibitory interactions, as diagrammed in Figure 4A. For a short duration above 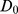, the activation probability increases linearly with increasing duration. As shown in Figure 4C, the rates of linear increase are dependent on the stimulus intensity (line shows linear fit, R^2^ = 0.997). As with the toggle switch, the priming delay 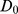 decreases exponentially with increasing stimulus intensity (Figure 4D, R ^2^ = 0.985).

### Advantages of linear activation

Previous studies have demonstrated that negative feedback reduces the effect of biochemical noise and linearizes dose responses [38]. The negative-feedback-mediated linearization of dose responses occurs at the individual cell level. Our preceding analysis suggests that population response can also be linearized the in the duration domain: bistable switches amplify expression variability in individual cells and linearize the population-level response to transient stimuli.

How is the linear response advantageous to a cell population, compared to nonlinear responses, to a cell population sensing transient stimuli? For monotonically increasing response functions 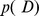 over a given range of signal durations, a linear response is equally sensitive to variability in the signal duration regardless of the value of 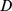. That is, because the derivative 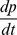 is constant, any fluctuation 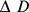 will result in the same change 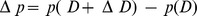, regardless of the value of 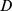. In this manner, the bistable decision serves to integrate a transient input signal, respond cumulatively to fluctuating environmental conditions, and neither “over-commit to” nor “withhold from” a signal-dependent alternate steady state.

**Figure 5:**
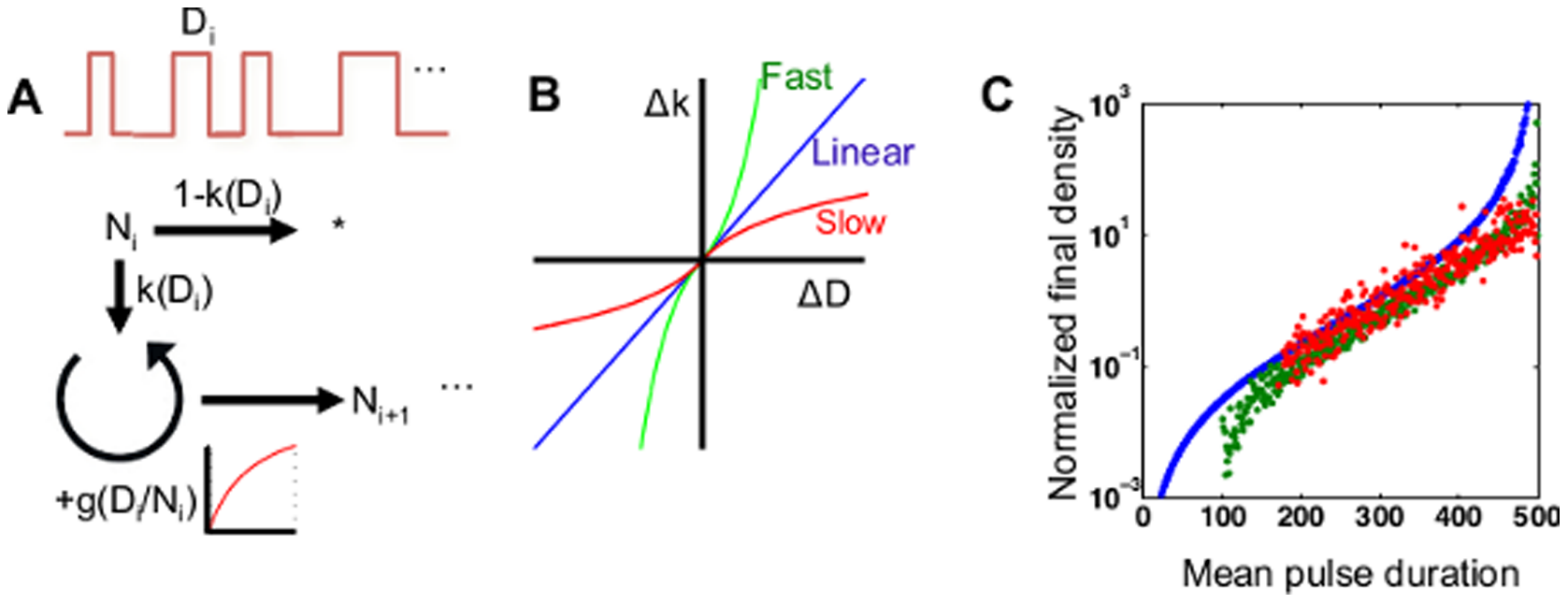
Linear increase in activation probability is a preferable allocation strategy. (A) A discrete bistable system allows comparison of various activation probability curves. In this model, successive pulses of unit intensity, mean duration *D*, and Gaussian-distributed deviation *ΔD_i_* are applied to a population. For each pulse, a fraction p(D+*ΔD*) *are* “activated” and proliferate. The rate of proliferation is assumed to be dependent on the signal intensity per individual ((*D+ΔD_i_*)*N_i_*), following a Monod function. The remaining inactivated subpopulation is eliminated (i.e. cell death). We note that the conclusions about relative fitness are not affected if the inactivated subpopulation is assumed to be quiescent (alive but non-growing). Successive pulses can be applied to simulate fluctuating environmental conditions. (B) Three characteristic activation probability curves as a function of signal duration. Population response can be grouped into one of three categories. A fluctuation in signal duration can result in a corresponding linear change in activation (blue line), or a change that is more sensitive (red line) or less sensitive (green line) than linear. Note that all three representative curves are defined over the same range of signal durations, and *k*(0) = 0, *k*(500) = 1 for all. (C) For each characteristic curve in (B), we plot the fold change after 1,000 pulses of a particular mean duration. A linear allocation strategy results in a larger fold change than fast and slow activation for 100% and 86% of signal durations, respectively. We note that this qualitative conclusion is true regardless of initial population size and number of pulses.

To illustrate this point, we construct a minimal model in which an isogenic population responds to a train of multiple pulsatile stimulatory signals (Figure 5A); this approach has previously been used to evaluated bistable populations [39]–[42]. These signals can be interpreted as a necessary resource (e.g. nutrients) for cells to grow and divide. That is, the duration of a pulse is proportional to the amount of resource made available to the population. The durations of these pulses are normally distributed around a mean value 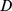, with 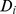 being the deviation for the 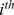 pulse. In each pulse, a fraction of the total population 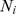 is stimulated to proliferate, and the rest are eliminated (i.e. cell death). This bifurcation represents a bistable event: each individual either survives or does not. As such, the surviving fraction, or the activation probability, is a function of the signal duration 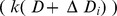. Furthermore, *we assume that the growth rate of surviving cells depends on the amount of resource per individual* (
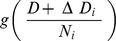
), following a Monod equation [43], [44]. We simplified this model by assuming that resources are divided equally among all members of the population, and that the input signal intensity is strong enough such that variability in its intensity is negligible. Here, it is important to note that our model assumes that resources, once allocated, are consumed and thus unavailable to other cells in the population, e.g. when a stimulus pulse corresponds to nutrient availability. However, this may not be true for all stimuli. For example, transcription factors and enzymes can freely diffuse between, and affect, multiple cells without being consumed or degraded.

In this scenario, how should populations respond to fluctuations in signal duration? That is, given a train of pulses with variable duration, what is the ideal strategy to maximize the population fitness? To examine this question, we compared three representative response strategies:

1. Linear activation (Figure 5B, blue line): a fluctuation in signal duration 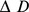 results in a linear change in the surviving fraction.
2. Fast activation (Figure 5B, green line): a fluctuation in signal duration 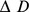 results in a stronger than linear change in the surviving fraction; a positive 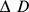 results in a greater increase, and a negative 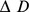 results in a greater decrease, in the surviving fraction when compared to linear activation. This strategy represents a “greedy” response, in that the over-allocation restricts the amount of resource available to each individual.
3. Slow activation (Figure 5B, red line): the opposite of fast activation; a fluctuation in signal duration Δ*D* results in a weaker than linear change in the surviving fraction; a positive 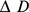 results in a lesser increase, and a negative 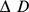 results in a lesser decrease, in the surviving fraction when compared to linear activation. This strategy represents a “cautious” response, in that under-allocation does not take full advantages of the resources available.

Here, we note that the curves shown in Figure 5B show *changes in activation probability*; the growth rate is fixed by the Monod equation, and the deviations can be linear, fast, or slow, as described above. We then simulated the effect of these different strategies on independent populations (please see Materials and Methods for detailed description). For each possible input signal duration, a train of variable pulses was used to stimulate the growth and/or decay of a population. We define fitness as the fold change between the final and initial population sizes. As shown in Figure 5C, a linear activation strategy outperformed (larger fold difference) both slow and fast activation for the majority of durations tested (100% and 86% of durations, as compared to slow and fast activation, respectively). These results suggest that the transition rate between bistable states can be optimized, especially in scenarios in which transition dynamics can affect overall fitness in opposing ways. We note that these conclusions also hold true if the inactivated subpopulation is assumed to be quiescent (alive but non-growing).

In the model examined here, fast activation appears beneficial in that more individuals survive the bistable decision; however, it results in lower per capita resource availability, and therefore a lower growth rate (overestimating the resource availability). Conversely, slow activation results in a higher growth rate due to higher per capita resource availability, but fewer individuals survive to take advantage of those favorable conditions (underestimating the resource availability). Clearly, there is a need to balance these interconnected effects. Linear activation is a happy medium that allows populations to utilize fluctuating conditions moderately, without being greedy or overly cautious.

## Discussion

Bistable decisions have been shown to underlie many cellular decisions, including differentiation and stress response. Previous studies have shown that pulsatile stimulation can be modulated to affect downstream gene expression in calcium-mediated signaling in yeast [45], NF-κB signaling in mammalian cells [46], and T cell stimulation [47]. These studies demonstrated that cell populations can multiplex different modes of information (signal intensity, duration, frequency etc.) to implement complex behaviors [48]. In many cases, these decisions are made on a single cell level; inherent biochemical stochasticity implies that the responses will vary within a population. In this context, it becomes important to understand how populations respond to non-saturating stimuli in the presence of cellular noise. Here we have demonstrated a characteristic linear dependence of activation probability on input signal duration in various stochastic bistable systems. Using a simple nonlinear feedback model, we showed that this activation probability can be mathematically predicted; we then demonstrated the same property in two increasingly complex systems, the toggle switch, and the Myc-Rb-E2F cell cycle network.

We propose that this deterministic duration-response linearity is an inherent feature of bistable systems, allowing populations to optimally respond to transient signals in a “real-time” manner, without *a priori* knowledge of signal duration. By allocating an appropriate number of individuals to each state, bistable networks could serve to ensure that populations take full advantage of favorable stimuli (e.g. increased nutrient levels), or avoid non-favorable factors (e.g. antibiotics). Notably, while positive feedback is generally thought to amplify variability, we show that bistable systems can process this individual uncertainty (at the single cell level) into a collective certainty (at the population level). Moreover, our results suggest that linear activation can be a better option than both excessively fast and prohibitively slow responses as a population survival strategy under fluctuating environment. Indeed, this linear response can be observed in a variety of scenarios. Recent work has demonstrated the emergence of linear response in T cell activation, wherein naïve T cells differentiate into helper T cells in response to antigen exposure [49]. As the antigen exposure duration is lengthened, the percentage of T cells transition to helper T cells increases linearly and then asymptotically approaches a maximum [47]. This differentiation decision is critical to generating a defensive immune response while minimizing the risk of autoimmunity [50]. Linear activation has also been observed in caspase-mediated apoptosis models, in which the fraction of dead cells increases linearly with the duration of treatment with an apoptosis inducer such as Apo2L/TRAIL after an initial priming delay [51]. These conclusions are analogous to the response we have described; further demonstrating that linear response can be generated by a diversity of bistable motifs.

## Materials and Methods

### Stochastic simulations

All stochastic simulations were implemented using the Gillespie approximation to the chemical Langevin equation, using custom Matlab scripts (see Supporting Information for detailed description).

### Linear regime fitting (toggle switch and Myc-Rb-E2F model)

For a given stimulus intensity, the duration giving 50% activation was interpolated. The immediately surrounding data points were used to fit a straight line: the slope of that line is the linear regime slope, and the priming delay is the x-intercept.

### Linear advantage simulations

A sequence of pulse durations was randomly generated, normally distributed with a given mean duration and variance equal to 10% of the mean. All populations were of the same initial size. For each pulse in the sequence, a fraction of the population (dictated by the particular allocation strategy used) multiplied. The remainder of the population was discarded. The growth rate was assumed to be a Monod function of the per capita resource level, that is, pulse duration normalized by current population size. This procedure was carried out with 1000 consecutive pulses, after which the fitness was calculated as the fold change between the final and initial population sizes. All simulations were implemented in Matlab.

## Supporting Information

Figure S1(A) The effect of autocatalysis. In the absence of stimulus (dotted line), the emergence and relative placement of steady states in the non-dimensional model can be tuned with the autocatalytic parameter “c”. For low values of this parameter, the only steady state is at 0. However, with sufficiently strong positive feedback, two nonzero steady states emerge (c_off_, blue points, and c_on_, red points). (B) Potential energy landscapes as a function of stimulus intensity. If the autocatalysis parameter “c” is chosen such that nonzero steady states are attainable, the potential energy landscape forms two wells corresponding to OFF and ON. Increasing stimulus intensity simultaneously leads to a shallower well at c_off_ and a deeper well at c_on_. This trend corresponds to an overall shift in the population from OFF to ON with increasing stimulus. Here we used *c* = 1.45, 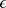 = 0.005, and *D*_e_ = 0.01.(TIF)

Figure S2 Determination of p_act_ from stochastic simulations of the positive-feedback model. Due to the presence of stochastic noise, replicates with identical initial conditions form distributions centered around two steady states. Time courses (left panel) of 10,000 simulations were separated into OFF and ON fractions based on the boundary given by the critical unstable concentration value (middle panel, dotted line). The ON fraction was then plotted as a function of stimulus duration (right panel). We used the same method to evaluate the positive-feedback model and the toggle switch model.
(TIF)

Figure S3 (A) Simulated E2F time courses in response to a pulse input with varying durations. For sufficiently strong stimuli (e.g. S = 5), increasing duration led to a characteristic adaptive response in E2F level (ON). The fraction of individuals exhibiting this adaptive response is a function of stimulus duration. (B) and (C) OFF and ON populations were delineated by the width of the pulsatile response. For each time course, width of the pulsatile response at the half maximum E2F level was determined. This metric resulted in a bistable distribution: the population centered at the higher mode was taken as the activated fraction.
(TIF)

Text S1 (DOCX)
