# Supplementary figures and images for: Linear Population Allocation by Bistable Switches in Response to Transient Stimulation
Source: PLoS One. 2014 Aug 20;9(8):e105408. doi: 10.1371/journal.pone.0105408 (PMC4139379; doi:10.1371/journal.pone.0105408)

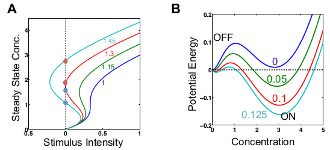

Supplement: Figure S1 — (A) The effect of autocatalysis. In the absence of stimulus (dotted line), the emergence and relative placement of steady states in the non-dimensional model can be tuned with the autocatalytic parameter “c”. For low values of this parameter, the only steady state is at 0. However, with sufficiently strong positive feedback, two nonzero steady states emerge (coff, blue points, and con, red points). (B) Potential energy landscapes as a function of stimulus intensity. If the autocatalysis parameter “c” is chosen such that nonzero steady states are attainable, the potential energy landscape forms two wells corresponding to OFF and ON. Increasing stimulus intensity simultaneously leads to a shallower well at coff and a deeper well at con. This trend corresponds to an overall shift in the population from OFF to ON with increasing stimulus. Here we used c = 1.45, = 0.005, and D e = 0.01. (TIF) [file pone.0105408.s001.tif]

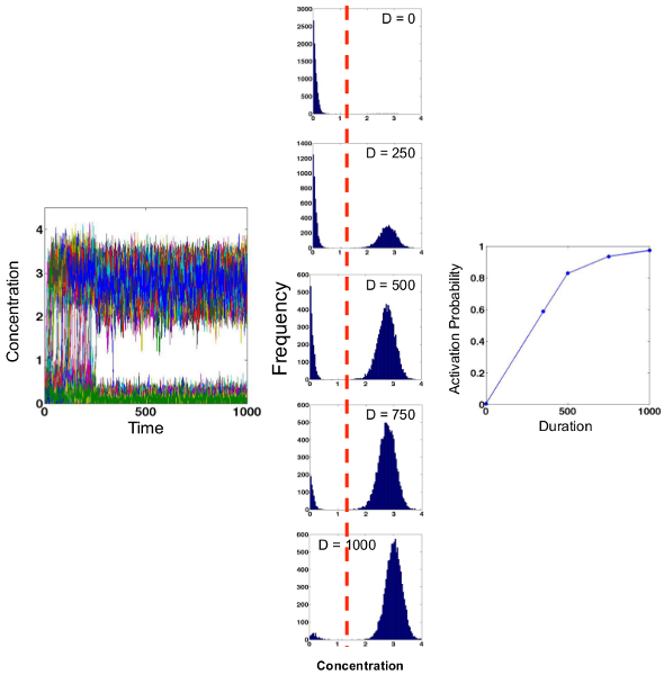

Supplement: Figure S2 — Determination of pact from stochastic simulations of the positive-feedback model. Due to the presence of stochastic noise, replicates with identical initial conditions form distributions centered around two steady states. Time courses (left panel) of 10,000 simulations were separated into OFF and ON fractions based on the boundary given by the critical unstable concentration value (middle panel, dotted line). The ON fraction was then plotted as a function of stimulus duration (right panel). We used the same method to evaluate the positive-feedback model and the toggle switch model. (TIF) [file pone.0105408.s002.tif]

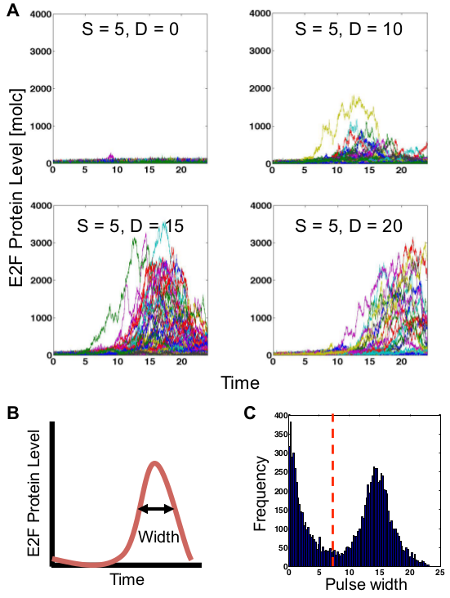

Supplement: Figure S3 — (A) Simulated E2F time courses in response to a pulse input with varying durations. For sufficiently strong stimuli (e.g. S = 5), increasing duration led to a characteristic adaptive response in E2F level (ON). The fraction of individuals exhibiting this adaptive response is a function of stimulus duration. (B) and (C) OFF and ON populations were delineated by the width of the pulsatile response. For each time course, width of the pulsatile response at the half maximum E2F level was determined. This metric resulted in a bistable distribution: the population centered at the higher mode was taken as the activated fraction. (TIF) [file pone.0105408.s003.tif]
