# Supplementary material for: Linear Population Allocation by Bistable Switches in Response to Transient Stimulation
Source: PLoS One. 2014 Aug 20;9(8):e105408. doi: 10.1371/journal.pone.0105408 (PMC4139379; doi:10.1371/journal.pone.0105408)
Supplement: Text S1 — (DOCX) [file pone.0105408.s004.docx]

**Supporting Information**

**Linear population allocation by bistable switches in response to transient stimulation**

Jaydeep K. Srimani^1*^, Guang Yao^2*^, John Neu^1^, Yu Tanouchi^1^, Tae Jun Lee^1^, and Lingchong You^1,†^

*^1^Department of Biomedical Engineering, Duke University, Durham, NC 27708, USA.*

*^2^Department of Molecular & Cellular Biology, University of Arizona, Tucson, AZ 85721, USA.*

* These authors contributed equally to this work.

^†^ Correspondence should be addressed to: L.Y. ([you@duke.edu](mailto:you@duke.edu))

**A bistable positive-feedback circuit**

Bistable systems can be defined as those that allow a species of interest (e.g. chemical species, protein numbers, etc.) to assume one of two stable states (e.g. concentrations). In response to stimulatory/inhibitory signals, the system state can be changed; regardless, when the signal is withdrawn, the current state of the system is maintained. These motifs are common in gene/protein networks, wherein they can function as decision-making modules. For example, bistable switches govern cell differentiation and proliferation and the production of virulence factors in many bacterial pathogens. Several mechanisms can give rise to bistability, including nonlinear positive feedback [[1](#_ENREF_1)], ßprotein titration [[2](#_ENREF_2),[3](#_ENREF_3)], and ultrasensitivity [[4](#_ENREF_4),[5](#_ENREF_5)]. Here, we use a simple cooperative system to look at population behavior. In this case, the synthesis of a species concentration of interest up-regulates its production, leading to a threshold-like behavior characteristic of a Hill function. From this biochemical perspective, we can specify a bistable system using a single ordinary differential equation (ODE) as follows:

| $\dot{x}=f\left( t \right)+a\left( x \right)-x$ | (S1) |
| --- | --- |

where $f(t)$ represents an external source signal that can be experimentally controlled and can potentially serve as a trigger for the switch, $a(x)$ represents a necessarily nonlinear, autocatalytic Hill term leading to bistability, and $-x$ denotes degradation of the species of interest. Here, the dimensionless time constant of the decay term is unity, indicating that the physical unit of time is the inverse of the decay time constant. In the simplest case, with $f\left( t \right)=0$, we can assume that the autocatalytic term is such that the unforced system has two stable equilibria (say $x_{off}<x_{on}$) separated by a critical point $x=x_{c}$. In this case, the end state of a particular trajectory $x(t)$ is dictated by the initial condition $x_{0}=x(0)$. If $x_{0}<x_{c}$, the system will approach one steady state (e.g. OFF), whereas if $x_{0}>x_{c}$, the system will approach the other steady state (e.g. ON).

Initially, we will impose a number of general constraints on this non-dimensionalized system, the better to mimic actual stochastic biological systems:

1. In the absence of external stimulus $f(t)$, extrinsic noise should induce a basal fraction of individuals to the ON state.
2. The rate of transition from ON to OFF (the ‘reverse’ direction), while non-zero, will follow a time constant much longer than the OFF to ON transition; therefore, there will be negligible ‘deactivation’ within the time scale of interest.

Without loss of generality, let us take the autocatalytic term $a\left( x \right)$ from Eq. S1 above as:

| $a\left( x \right)=\frac{8}{3}c\frac{x^{2}}{3+x^{2}}$ | (S2) |
| --- | --- |

Using this Michaelis-Menten form with appropriate parameter choices ensures bistability. Here, we have chosen the pre-factor and half-maximal values such that $a(x)$ has its inflection point corresponding to the maximum of $\frac{da}{dx}$ at $x=1$. Therefore, ${\frac{da}{dx}}_{|x=1}=c$. Bistability in the kinetics dictated by Eq. S1 are shown in the following bifurcation diagram (Figure S1A). Here, we have set $\dot{x}=0$ to show the steady states of $x$ as a function of constant stimulus $f$. Each trajectory in Figure S1A represents this relationship for a particular value of the constant $c$. A particular steady state value of $x$ is linearly stable if $\frac{d(x-a(x))}{dx}>0$; accordingly, the portions of the trajectories with positive slopes represent stable branches. Conversely, the portions with negative slope are unstable branches. If $c<1$, the graph of $x$ vs $f$is monotonically increasing; there is a unique stable point. For $c=1$, a vertical tangent occurs at $\left( x,f \right)=(1,\frac{1}{3})$. For each $c>1$ condition, then, we have a bistable range of $f$, separated by stable branches where the system is either OFF (lower branch) or ON (upper branch); the points at which the bistable range meets either the stable or unstable branch are saddle-node bifurcations. We note that the ON saddle-node bifurcation crosses the $f$ axis for $c=\frac{3\sqrt{3}}{4}\approx1.299$. This means that for $c<\frac{3\sqrt{3}}{4}$, there will be no ON state in the absence of stimulus ($f=0$); conversely, for $c>\frac{3\sqrt{3}}{4}$, there exists an ON state with $f=0$.

These trajectories correspond to situations in which a subpopulation will be activated even in absence of stimulus, satisfying condition (i) from above. We have chosen $c= 1.45$, which corresponds to a metastable ON state; although a subpopulation may be activated in the absence of stimulus (due to stochastic noise), all individuals will settle at the OFF state given an infinitely long time span. In this work, we are interested in the population response to a transient square wave signal $f(t)$; that is, $f\left( t<D \right)=k>0, f\left( t>D \right)=0$ for some arbitrary signal duration $D$.

**Deriving an analytical solution for activation probability**

In practice, the deterministic kinetics given by the ODE in Eq S2 are perturbed by random noise. Here, we consider two sources of such noise: a chemical noise (intrinsic to $x(t)$ itself), and an extrinsic component due to the inherent randomness of the system environment. Because $x(t)$ is a protein concentration, and changes therein are discrete events, we model the noisy dynamics using Poisson processes; therefore, at any time, a population will form a distribution with positive variance.

We can use a Fokker-Planck formulation to examine the behavior of this system in the presence of stochastic noise. The dependent state variable is the ensemble probability density $p(x)$. Specifically, in a given population, the fraction of individuals with concentrations $x(t)$ in the range $(x,x+dx)$ is $p\left( x,t \right)dx$. The dimensionless Fokker-Planck equation (FPE) for the system can be simply written as a convection-diffusion partial differential equation (PDE) in x-space as follows, where $j$ denotes the flux term, and subscripts indicate first partial derivatives:

| $p_{t}+j_{x}=0$ $j=\left( a\left( x \right)-kx+f \right)p-\left\{ \left( D_{E}+\frac{1}{2V}\left( a\left( x \right)+kx+f \right) \right)p \right\}_{x}$ | (S3) |
| --- | --- |

Here, the first term in $j$ represents the convection of probability in accordance with the deterministic kinetics. The second term represents diffusion of probability due to the two aforementioned sources of random noise. Specifically, $D_{E}$ represents external noise; the remaining terms represent the probability flux associated with chemical noise. Nondimensionalization yields the following form, where $\epsilon$ scales the intrinsic, state-dependent noise component:

| $j=\left( a\left( x \right)-x+f \right)p-\left\{ \left( D_{E}+\epsilon(a\left( x \right)+x+f) \right)p \right\}_{x}$ | (S4) |
| --- | --- |

In chemistry, “detailed balance” refers to equilibria in which forward and backward reaction rates are exactly balance, and there is no net changes in species concentrations. In an FPE, the analogous situation occurs when the probability flux vanishes identically. If the state space of independent variables (here $x(t)$) is one-dimensional, we can always find equilibria characterized by detailed balance. If we set $j=0$, the solutions $p\left( x \right)$ are proportional to the following Boltzmann factor:

| $p\left( x \right)\propto e^{\frac{-U(x)}{D_{E}}}$ | (S5) |
| --- | --- |

Where $U(x)$ is the effective potential in the concentration space. This satisfies the following relationship:

| $\dot{U}\left( x \right)=\frac{x-a\left( x \right)-f-\epsilon(\dot{a}\left( x \right)+1)}{1+\frac{\epsilon}{D_{E}}(a\left( x \right)+x+f)}$ | (S6) |
| --- | --- |

The Boltzmann factor, as a function of $x$, has maxima at the minima of $U(x)$. In the case of zero intrinsic noise ($\epsilon=0$), Eq S6 reduces to the conditions for a stable critical point in the deterministic system.

As shown in Figure S1B, the potential landscape, $U(x)$ vs $x$, consists of two wells separated by a barrier, reflecting the stable points of the system and the perturbation required to transition between them. The range of stimulus $f$ shown is within the aforementioned bistable range. For each landscape, the wells are given by the minima of $U(x)$: we will denote these minima as $U_{off}=U\left( {f,x}_{off} \right)$ and $U_{on}=U(f,x_{on})$, where $U_{on}>U_{off}$. These minima are separated by an energy barrier; we will define $E=U\left( x_{c} \right)-U_{off}$. This is the energy required to transition from the OFF state to the ON state. With no stimulus ($f=0$), the OFF state is favored; as $f$ increases, the ON state becomes increasingly favored.

We are interested in transient stimuli; in these situations, a population will encounter two landscapes. Initially, with the application of stimulus, the nonzero stimulus will yield a landscape more permissive towards activation, allowing a fraction of individuals to overcome a relatively low $E$. When stimulus is removed, the landscape reverts, and activation becomes significantly less likely (please refer to Figure 1C). We are interested in the fraction $p_{act}(t)$, which we define to be the proportion of a population that is activated during a transient stimulus of length $t$. That is, the fraction that surpasses $x_{c}$ within the interval $t<D$ with nonzero stimulus. The rate of transition from OFF to ON is ultimately dependent on $f$. The predicted rate constant based on asymptotic analysis of the Fokker-Planck equation in the small noise limit $\frac{E}{D_{e}}$ is as follows:

| $r:=\frac{1}{2\pi}\sqrt{\ddot{U}_{off}\left\vert\ddot{U}_{c} \right\vert}e^{\frac{E}{D_{e}}}$ | (S7) |
| --- | --- |

In general, then, $p_{act}\left( t \right)$ satisfies the following ODE:

| $\dot{p_{act}}\left( t \right)=r\left( 1-p \right)$ | (S8) |
| --- | --- |

It is easily seen that the ODE satisfied by:

| $p_{act}\left( t \right)=1-e^{-rt}$ | (S9) |
| --- | --- |

We note that for weak stimuli, which correspond to small values of $r$, the activation probability increases linearly for sufficiently short stimulus durations ($\frac{dp_{act}}{dt}\approx r$).

**Noise Implementation and Stochastic Simulation Algorithm**

We use a Langevin approach [[6](#_ENREF_6)] to study the effects of intrinsic and extrinsic noise in all simulations discussed. Specifically, we use the form shown below:

| $\frac{dX_{i}}{dt}=\sum_{j=1}^{M} v_{ji}a_{j}[X(t)]+\sigma\sum_{j=1}^{M} v_{ji}a_{j}^{1/2}\left[ X\left( t \right) \right]\Gamma+\delta\omega$ | (S10) |
| --- | --- |

where $X_{i}(t)$ represents the population of species *i* at time *t*, and $X(t)$ is the complete system state at time  *t*. Therefore, the system temporally evolves according to the rates $a_{ji}[X(t)]$ with the inherent changes in molecular number given in $v_{ji}$, $\Gamma$ and $\omega$ are temporally uncorrelated, statistically independent normal Gaussian noises. In this formulation, the first term on the RHS represents deterministic kinetics, the second term represents reaction-dependent (intrinsic) noise, and the third term represents reaction-independent (extrinsic) noise. The factors $\sigma$ and $\delta$ are scaling factors that can be precisely adjusted to tune the levels of intrinsic and extrinsic noise, respectively. All reaction rate parameters and species concentrations were converted to molecular numbers as appropriate.

For each of the models analyzed, we initialize 10,000 replicates, which can be thought of as iso-clonal, synchronized, independent individuals. We then track their temporal evolution using an iterative forward-stepping method, which can be summarized as follows. The derivative term is taken from the Langevin formulation above:

| $A\left( t \right)=A\left( t-\Delta t \right)+\left[ \frac{dA}{dt} \right]_{t}\Delta t$ | (S11) |
| --- | --- |

Using this forward Euler approach, we generated time series data and used bimodality in end time concentration levels as the criterion for bistability. Figure S2 shows a representative example of the non-dimensional cooperative switch. As shown, the same critical level $x_{*}$ was used for all dosing parameter combinations. All models were implemented and analyzed using custom MATLAB scripts.

**A toggle switch model**

We use a variant of the toggle switch model originally derived by Gardner et al. [[7](#_ENREF_7)]. In this model, nonlinear mutual inhibition between two species creates a bistable landscape. The deterministic ODEs and rate parameters used are as follows:

| $\frac{d[A]}{dt}=k_{0}+f\left( [S] \right)+\frac{k_{A}}{K_{1}+\left[ B \right]^{n}}-d_{A}[A]$ | (S12) |
| --- | --- |
| $\frac{d[B]}{dt}=\frac{k_{B}}{K_{2}+\left[ A \right]^{n}}-d_{B}[B]$ | (S13) |

| **Parameter** | **Value** |
| --- | --- |
| k_0_ (Basal level production) | 1E-5 [μM/hr] |
| k_A_ (Synthesis rate of A) | 0.7 [μM/hr] |
| K_1_ (Half maximal level of A) | 1.66E-4 [μM] |
| k_b_ (Synthesis rate of B) | 1 [μM/hr] |
| K_2_ (Half maximal level of B) | 1.66E-4 [μM] |
| d_A_ (Degradation rate of A) | 0.2 [1/hr] |
| dB (Degradation rate of B) | 0.95 [1/hr] |
| σ (Intrinsic noise scale factor) | 1 |
| δ (Extrinsic noise scale factor) | 1 |

**Myc-Rb-E2F Model**

The Myc-Rb-E2F pathway has been extensively studied in connection with mammalian cell cycle control and cell fate decisions [[8](#_ENREF_8),[9](#_ENREF_9)]. When exposed to appropriate growth factors (serum, Myc, etc), E2F family proteins are upregulated, exhibiting a peak and decline behavior characteristic of adaptive response. This transient increase in E2F levels in turn regulates hundreds of downstream genes that lead to DNA replication and eventual cell division. As such, dysregulation of this network has been implicated in many cancer types, and constitutes an active area of study [[10-12](#_ENREF_10)]. Here, we use the Myc-Rb-E2F pathway (Figure 4A in the main text) as a case study; we take the model equations and rate parameters from the literature [13[13](#_ENREF_13),[14](#_ENREF_14)], and apply the simulation method described above. As shown in Figure S3A, in response to transient serum stimulation, a subpopulation of individuals will exhibit upregulation of E2F. Furthermore, this subpopulation will become more prevalent as the pulse lengthens, as can be seen in the progressive panels. We detect the accompanying adaptive response numerically and use the width at the half-maximal E2F level as the distinguishing metric between inactivated (small width) and activated (large width) individuals (Figure S3B); this metric yields a bimodal distribution (Figure S3C) which we use to separate the activated fraction.

**Initial lag in the toggle-switch and Myc-Rb-E2F models**

As shown in Figure 3B and Figure 4B in the main text, simulation results for both the toggle switch and Myc-Rb-E2F network show an initial lag phase, wherein for low stimulus durations, the activation probability does not increase appreciably. Subsequently, for sufficiently long durations, the activation probability appears to behave according to the previously described exponential relationship. This is not unexpected; intuitively, weak stimuli may fail to trigger transitions between steady states.

We propose that this effect is more noticeable for the toggle switch and Myc-Rb-E2F networks due to the presence of negative feedback regulation: the toggle switch modeled here is based on NF interactions as discussed in the main text. In the mammalian cell cycle model, titration of E2F by the protein Rb imposes a significant inhibition of E2F levels. In these cases, negative feedback sets a barrier to activation that must be overcome, hence the initial lag. In contrast, the cooperative model contains no such NF interactions. There will still be an initial lag in the cooperative system (due to degradation at low concentration), but evidently it is on a much smaller timescale than the minimum stimulus durations sufficient for activation.

**References**

1. Ferrell J, James E (2002) Self-perpetuating states in signal transduction: positive feedback, double-negative feedback and bistability. Current Opinion in Cell Biology 14: 140-148.

2. Buchler NE, Cross FR (2009) Protein sequestration generates a flexible ultrasensitive response in a genetic network. Molecular Systems Biology 5.

3. Chen D, Arkin AP (2012) Sequestration-based bistability enables tuning of the switching boundaries and design of a latch. Molecular Systems Biology 8: 1-7.

4. Huang C-Y, Ferrell JE (1996) Ultrasensitivity in the mitogen-activated protein kinase cascade. Proceedings of the National Academy of Sciences 93: 10078-10083.

5. Markevich NI (2004) Signaling switches and bistability arising from multisite phosphorylation in protein kinase cascades. The Journal of cell biology 164: 353-359.

6. Gillespie DT (2000) The chemical Langevin equation. The Journal of Chemical Physics 113: 297.

7. Gardner TS, Cantor CR, Collins JJ (2000) Construction of a genetic toggle switch inEscherichia coli. Nature 403: 339-342.

8. Nevins JR (1992) E2f - a Link Between the Rb Tumor Suppressor Protein and Viral Oncoproteins. Science 258: 424-429.

9. Chellappan SP, Hiebert S, Mudryj M, Horowitz JM, Nevins JR (1991) The E2f Transcription Factor Is a Cellular Target for the Rb Protein. Cell 65: 1053-1061.

10. Nevins JR (2001) The Rb/E2F pathway and cancer. Human Molecular Genetics 10: 699-703.

11. Jung H, Hsiung B, Pestal K, Procyk E, Raulet DH (2012) RAE-1 ligands for the NKG2D receptor are regulated by E2F transcription factors, which control cell cycle entry. Journal of Experimental Medicine 209: 2409-2422.

12. Molenaar JJ, Koster J, Ebus ME, van Sluis P, Westerhout EM, et al. (2012) Copy number defects of G1‐Cell cycle genes in neuroblastoma are frequent and correlate with high expression of E2F target genes and a poor prognosis. Genes, Chromosomes, and Cancer 51: 10-19.

13. Lee TJ, Yao G, Bennett DC, Nevins JR, You L (2010) Stochastic E2F activation and reconciliation of phenomenological cell-cycle models. PLoS Biology 8: e1000488.

14. Yao G, Tan C, West M, Nevins JR, You L (2011) Origin of bistability underlying mammalian cell cycle entry. Molecular Systems Biology 7: 1-10.
